# The Role of Nutraceuticals and Probiotics in Addition to Lifestyle Intervention in the Management of Childhood Obesity—Part 1: Metabolic Changes

**DOI:** 10.3390/nu17101630

**Published:** 2025-05-09

**Authors:** Maria Elisabeth Street, Federica Casadei, Erika Rita Di Bari, Francesca Ferraboschi, Anna Giuseppina Montani, Anna-Mariia Shulhai, Susanna Esposito

**Affiliations:** 1 Department of Medicine and Surgery, University of Parma, 43126 Parma, Italy; federica.casadei@unipr.it (F.C.); erikarita.dibari@unipr.it (E.R.D.B.); francesca.ferraboschi@unipr.it (F.F.); annagiuseppina.montani@unipr.it (A.G.M.); annashulhai@gmail.com (A.-M.S.); susannamariaroberta.esposito@unipr.it (S.E.); 2 Unit of Paediatrics, P. Barilla Children’s Hospital, University Hospital of Parma, 43126 Parma, Italy

**Keywords:** obesity, nutraceuticals, probiotics, metabolic syndrome, dyslipidemia, insulin resistance, diabetes mellitus

## Abstract

Childhood obesity is a growing global health issue. Its rising prevalence is linked to genetic, environmental, and lifestyle factors. Obesity in children could lead to different comorbidities and complications with an increased risk of metabolic disorders, such as insulin resistance, dyslipidemia, type 2 diabetes mellitus (T2DM), and metabolic dysfunction-associated steatotic liver disease (MASLD). First-line treatment involves dietary modifications and lifestyle changes; however, adherence is often poor and remains a significant challenge. Pharmacotherapy, while a potential option, has limitations in availability and can cause side effects, leading to growing interest in alternative treatments, such as nutraceutical compounds. Derived from natural sources, these compounds have different anti-inflammatory, antiallergic, antioxidant, antibacterial, antifungal, neuroprotective, antiaging, antitumor, insulin-sensitizing, glucose, and lipid-lowering effects. This review describes commonly used nutraceutical compounds, such as omega-3 fatty acids, vitamin D, polyphenols (such as resveratrol and curcumin), berberine, white mulberry leaves and others, and pre- and probiotics in the management of obesity, evaluating the evidence on their mechanisms of action and efficacy in metabolic comorbidities. The evidence suggests that the integration of nutraceuticals into the diet may positively influence body mass index, glucose metabolism, lipid profiles, and gut microbiota composition and reduce inflammation in obese individuals. These effects may provide future practical guidance for clinical practice, contribute to metabolic health improvement, and potentially prevent obesity-related complications. In this first part, we discuss the effects of nutraceutical compounds on insulin sensitivity and insulin resistance, T2DM, dyslipidemia, and MASLD in addition to diet and lifestyle interventions.

## 1. Introduction

Childhood obesity is currently one of the most significant global health challenges. UNICEF and the World Health Organization (WHO) estimates reported that in 2022 overweight and obesity were present in 3.7 million children (5.6%) under 5 years of age worldwide [1]. This is a major concern, as this epidemic is associated with many adverse health outcomes since obese subjects are at high risk of developing severe comorbidities, most grouped under the definition of metabolic syndrome [2,3].

Metabolic syndrome (MetS) includes a cluster of cardiometabolic risk factors, such as visceral obesity, hypertension, dyslipidemia, and impaired glucose metabolism, that are associated with an increased risk of developing cardiovascular disease (CVD) and type 2 diabetes mellitus (T2DM). Although there is agreement on the distinctive features of MetS in adults, no agreed international diagnostic criteria exist for the pediatric population. In addition, MetS is also associated with metabolic dysfunction-associated steatotic liver disease (MASLD), which links the pathophysiology of fatty liver with metabolic dysfunction and insulin resistance. MASLD may progress into steatohepatitis, cirrhosis, and hepatocellular carcinoma [4,5]. Due to the difficulty of defining MetS in childhood, the current clinical practice approach is to address single co-morbidities and cardiometabolic risk factors [2,3,6,7], which should be considered by physicians.

Obesity-related health problems present at an increasingly early age and tend to progress into adulthood. Therefore, early identification and treatment of risk factors are crucial to prevent chronic diseases [8].

The first line of treatment for obesity consists of a multidisciplinary lifestyle intervention, including behavioral and dietary changes and regular physical activity. However, compliance with lifestyle adjustments is usually poor, fat loss is often only temporary, and it is quite common to gain weight after long-term treatment. Pharmacotherapy currently can be used in selected patients or severe cases, but it is often associated with side effects, particularly gastrointestinal issues [9]. Thus, nutraceutical interventions have gained interest as complements to lifestyle interventions and sometimes as effective alternatives to some pharmacological treatments. These bioactive compounds are derived from natural sources and have both nutritional value and pharmacological properties [10].

To date, several studies are available reporting the safety and effectiveness of nutraceutical products. The biological benefits rely mostly on phytochemicals, terpenoids, limonoids, phytosterols, polyphenols, isoflavonoids, fibers, and unsaturated fatty acids. Nutraceuticals act mainly as anti-inflammatory, antiallergic, antioxidant, antibacterial, antifungal, neuroprotective, antiaging, and antitumor agents [9,10,11,12]. Some of these compounds could be used for obesity and MetS treatment in children and adolescents [2,12]. Gut microbial modulation through the use of pre- and probiotics also seems to serve as a new therapeutic strategy to fight metabolic comorbidities associated with obesity and MetS [12]. In relation to metabolic disorders, a complex connection between oxidative stress and inflammation determines a cascade of events that significantly contribute to the advancement of comorbidities; thus, antioxidant and anti-inflammatory agents such as polyphenols, omega-3 fatty acids, and vitamins E and A are of interest for mitigating these dysregulations [10]. Many nutraceuticals have been proven to improve insulin sensitivity with moderate lipid-lowering action [13].

While several studies have explored the use of nutraceuticals in pediatric obesity, the available evidence is often limited to specific comorbidities or age groups. No comprehensive narrative review has synthesized the existing data across all major comorbidities associated with pediatric obesity. Summarizing current findings is essential to identify knowledge gaps, guide clinical decision-making, and inform future research directions.

In this narrative review, we describe nutraceuticals, probiotics and bioactive compounds that have been shown to have effects on obesity and their mechanisms of action, first on weight management and insulin sensitivity, and then in single comorbidities. Based on the current scientific evidence, the benefits and limitations of single nutraceutical compounds are presented with the available scientific support for clinical practice guidance.

## 2. Methods

Studies were identified through a comprehensive search across MEDLINE, PubMed, and Google Scholar. Relevant studies highlighting the impact of nutraceutical supplementation on obesity/overweight and related complications were selected using the following keywords: “pediatric”, and/or “child“, and/or “children”, and/or “childhood”, and/or “adolescent”, and “obesity”, and/or “overweight”, and/or “pediatric obesity”, and/or “appetite regulation”, and/or “satiety ”, and/or “dyslipidemia”, and/or “cholesterol“, and/or “triglycerides”, and/or “metabolic syndrome”, and/or “insulin resistance”, and/or “ insulin”, and/or “ insulin sensitivity”, and/or “diabetes mellitus” AND “nutraceuticals”, and/or “dietary supplements”, and/or “anti-obesity agents”, and/or “vitamins”, and/or “vitamin D”, and/or “vitamin E”, and/or “antioxidants”, and/or “phytosterols”, and/or “flavonoids” and/or “polyphenols”, and/or “probiotics”, and/or “prebiotics”, and/or “PUFA”, and/or “omega 3”, and/or “olive oil, and/or “green tea”, and/or “policaptil gel retard”, and/or “inositols”, and/or “ alpha lipoic acid”. These keywords were differently matched to obtain the search strings. For this review, systematic reviews, meta-analyses, randomized controlled clinical trials, controlled clinical trials, original articles, and case series were taken into consideration. All considered articles were published in English. The search was performed in the databases from 1993 to 2024. Whenever studies were missing for the age group 0–18 years, we considered studies in adults with findings that could be extended potentially to childhood. Both in vivo and in vitro studies were additionally considered for single compounds of greater interest. A thorough analysis and subsequent synthesis of the literature gathered from the search were conducted and are presented below. In part 1, we separately considered evidence on weight management in general, then on insulin sensitivity and on single metabolic comorbidities such as insulin resistance, type 2 diabetes mellitus, dyslipidemia and metabolic dysfunction-associated steatotic liver disease as the most commonly reported obesity-related metabolic complications.

## 3. Management of Weight and Regulation of Insulin Sensitivity

### 3.1. Diet and Lifestyle Intervention as the Mainstay

Substantial lifestyle modification is recommended for the treatment of obesity to achieve a balance between energy expenditure and caloric intake and a gradual but progressive reduction in body mass index (BMI) [3,14,15].

Diet therapy is crucial in the management of pediatric obesity [15]. Dietary interventions aim to reduce energy intake and improve eating habits, considering personal and cultural preferences and routines [15,16]. Dietary approaches, such as the ‘non-restrictive model’, which encourages the intake of low-fat, nutrient-dense foods, or the ‘traffic light diet’, which classifies foods by calorie density, and low glucose index diets, which encourage the intake of foods such as fruit, non-starchy vegetables, whole grains, meat, and fish at the expense of sugary drinks and sweets, have demonstrated benefits in reducing BMI and improving anthropometric parameters [15,16]. The ‘mindful eating’ approach aims to raise awareness of food choices and has proven to be effective in preventing obesity. The Mediterranean diet and the DASH (Dietary Approaches to Stop Hypertension) diet have shown benefits in improving obesity-related comorbidities [16] and have beneficial effects on obese patients with cardiovascular risk factors. Researchers have demonstrated the efficacy of the Mediterranean diet, rich in polyunsaturated fatty acids (PUFAs), fiber, flavonoids, and antioxidants, even in obese children [17].

In a randomized clinical trial, the low glycemic index diet was associated with a significant reduction in fasting plasma insulin and Homeostatic Model Assessment for Insulin Resistance (HOMA-IR) in obese children, compared with controls [18], and children with obesity and impaired glucose metabolism can benefit from low glycemic index diets [16]. Calculating the insulin index in diets is considered to be useful in decreasing hyperinsulinemia. A randomized, single-blind, and crossover trial in insulin-resistant obese adolescents showed that a low glycemic index-low insulin index meal was associated with a reduction in insulin response and feeling of hunger compared with low glycemic index-high insulin index meals [19]. Researchers showed that a low-calorie diet, with increased protein intake and reduced carbohydrate consumption, could lower BMI and improve metabolic markers, including insulin, HOMA-IR, and even blood pressure [20].

It is important to mention that physical exercise is crucial to prevent complications and has a positive impact on insulin resistance in obese pediatric patients [21]. The WHO recommends that all children and adolescents should be engaged daily for at least one hour in moderate-to-vigorous physical activity; additionally, time spent in sedentary behaviors should be limited to less than two hours a day [7]. Inactivity negatively impacts cardiorespiratory or muscular fitness, leading to unfavorable measures of adiposity, lipids, metabolic parameters, and cardiovascular risk. Children with obesity experience difficulties in beginning and maintaining physical activity programs since they present impaired motor skills due to excessive body weight, acquire negative feelings related to physical activity practice, and create a vicious circle in which sedentary habits become predominant. Nevertheless, adolescents with obesity showing a more active lifestyle have a better health-related quality of life compared to their inactive peers [7]. Exercise interventions should be tailored to the physical and psychological limitations related to obesity to improve physical self-esteem, physical activity pleasure, and long-term physical activity adherence. Aerobic or combined exercises (aerobic and resistance) seem to be the most effective modalities to reduce body fat, control obesity, and related cardiometabolic complications [7]. The Italian Society for Pediatric Endocrinology and Diabetology “childhood obesity study group” has recently recommended that exercise should be scheduled into three weekly sessions and should progressively reach the goal of 60 min each. Sedentary time should be gradually replaced by a similar amount of physical activity to meet at least 60 min/day of active exercising. Walking, remote physical activity, and exergaming might be considered supplemental tools for fighting inactivity [7].

Anaerobic training was also shown to effectively reduce insulin resistance as well as general and visceral adiposity in obese or overweight children, independent of race or sex [22]. Moreover, aerobic exercise in addition to resistance training was more effective than resistance training alone in improving visceral adiposity and metabolic profile in obese adolescents, also achieving improved insulin sensitivity [23]. The high-intensity interval training appeared to reduce fasting insulin, though not significantly. In contrast, continuous moderate-intensity exercise training has been shown to significantly reduce the insulin sensitivity index, HOMA-IR [24]. Additionally, physical exercises also modulate adipokine levels in obese children [25]. Specifically, exercise would seem to increase serum adiponectin, an important regulator of insulin sensitivity, and reduce leptin, a pro-inflammatory marker associated with insulin resistance [26,27].

In conclusion, diet and lifestyle interventions remain the cornerstone of pediatric obesity management, offering a foundational approach to long-term metabolic health. Nutritional strategies such as low-glycemic index diets, the Mediterranean diet, and mindful eating alongside structured physical activity have demonstrated significant benefits in reducing BMI, improving insulin sensitivity, and modulating appetite-regulating hormones. Despite barriers to adherence, particularly among children facing psychosocial and physical challenges, individualized and culturally sensitive interventions, coupled with family and behavioral support, can greatly enhance outcomes.

### 3.2. Evidence for the Use of Nutraceutical Compounds

The following paragraph reports knowledge and evidence on nutraceutical compounds that are recognized to have weight-lowering effects besides metabolic effects related to insulin sensitivity, blood glucose, and lipid levels.

Policaptil Gel Retard^®^ (PGR) is composed of polysaccharide macromolecules derived from raw materials rich in fibers (cellulose, glucomannan originated from *Amorphophallus konjac*, *Opuntia ficus indica*, chicory root from *Cichorium intybus*, *Linum usitatissimum*, *Tilia platyphyllos* flowers and bracts, and freeze-dried mallow root mucilage originated from *Althaea officinalis*). In addition, it contains sweet orange (0.2%), lemon (0.1%), essential oils, and a natural orange flavor (2.2%). Fibers with both water-soluble and insoluble properties can form gels that interact with dietary components like lipids and sugars due to their high water-binding and swelling capacities [28,29]. Eating polysaccharide macromolecules before meals forms a gel that slows carbohydrate and fat absorption. It also reduces appetite in both obese and non-obese subjects by extending colon transit time, stimulating satiety hormones and endogenous GLP-1 secretion, and promoting lasting fullness after eating [30]. A study in obese mice on a high-fat diet described improved metabolic dysfunction; the treatment enhanced glucose tolerance and insulin sensitivity, restored insulin-like growth factor binding protein (IGFBP) 2 levels, and reduced serum triglyceride levels. Moreover, a significant reduction in body weight was observed, unrelated to food intake, highlighting overall beneficial effects [28]. In a double-blind clinical trial in 46 obese children, the intake of two tablets (1450 mg/day) of PGR 20 min before meal ingestion was able to reduce postprandial triglyceride and ghrelin serum levels, with a significant reduction in appetite during the first 240 min of the postprandial phase compared to placebo [30]. A recent longitudinal, randomized, double-blind clinical trial conducted in 133 hyperinsulinemic obese children and adolescents showed that the administration of three tablets (2175 mg/day) of PGR before the two main meals for one year, in addition to a low glycemic index diet, led to a 22.4% reduction in BMI, decreased glycated hemoglobin (HbA1c) levels and acanthosis nigricans, and improved glucose metabolism and insulin sensitivity among children [29].

One study compared the effect of metformin combined with PGR vs. metformin alone in 129 children and adolescents with obesity and MetS. Researchers observed significantly improved weight loss, insulin sensitivity, and reduced adiposity after adding PGR to the base compared to metformin alone [31]. These results were also confirmed by studies in adults. A recent study showed, in fact, that taking PGR 30 min before the two main meals (4350 mg/day) was as effective as metformin (1500–2000 mg/day) in improving body weight, glycemic control, and insulin sensitivity, with PGR superiority in terms of both serum lipid-lowering effects and tolerability [32]. Nevertheless, these results need to be confirmed by additional studies that should include larger sample sizes and longer follow-up periods.

White mulberry leaves (*Morus alba*) have been widely used since ancient times in Asian countries as medicinal plants because of their biological and therapeutic properties in metabolic disorders such as diabetes and obesity. White mulberry leaf has been known to contain multiple active phytochemical constituents, including alkaloids, benzofurans, coumarins, flavonoids, phenolic acids, polysaccharides, and terpenoids [33]. 1-deoxynojirimycin (1-DNJ), a potent inhibitor of intestinal α-glucosidase, is the main active alkaloid with widely recognized hypoglycemic effects. Moreover, 1-DNJ has the capacity to suppress disaccharide catabolic enzyme activity, slow the breakdown of disaccharides into monosaccharides during metabolism, and delay the generation and absorption of glucose, thus lowering blood sugar levels, making it a potential antihyperglycemic medicine [33,34]. Flavonoids, such as rutin, quercetin, isoquercitrin, luteolin, phenolic acids, and polysaccharides, are natural antioxidants, all of which have research evidence and potential applications [34]. In vivo and in vitro evidence has shown that white mulberry leaf can improve insulin sensitivity and has lipid-lowering and antioxidant properties besides the hypoglycemic effect. Therefore, mulberry leaf has great potential as an anti-obesity agent [35].

In animal studies, the administration of *Morus alba* extract has shown effects on appetite control. In diet-induced obese mice treated with high doses of *Morus alba* extract (250 and 500 mg/kg) for 7 weeks, a statistically significant body weight loss and dose-dependent reduction in food intake were observed [36]. *Morus alba* contains compounds that have been reported to have an overall inhibitory effect on the synthesis and accumulation of lipids. It may inhibit gastrointestinal lipase activity with subsequent reduction in fat absorption, promote browning of white adipose tissue, reduce inflammation and lipid oxidation, and reduce adipocyte differentiation through suppression of key transcription factors and inflammatory enzymes [34]. Specifically, long-term administration of *Morus alba* extract decreased body weight and adiposity and regulated hepatic lipid accumulation in diet-induced obese mice [37]. In high-fat diet-induced obese mice, *Morus alba* inhibited lipid synthesis and promoted lipolysis, with an inhibition of total serum cholesterol levels and triglyceride accumulation in liver cells and elevation of adiponectin mRNA expression in adipocytes in a dose-dependent manner [38]. In another study, treatment with *Morus alba* (from a low dose of 133 to a higher dose of 666 mg/kg) for 12 weeks significantly improved lipogenesis and positively regulated markers associated with lipolysis, such as lipoprotein lipase, in obese mice [37]. In vitro, the 1-DNJ agent suppressed adipogenesis during the differentiation of white preadipocytes and promoted the transition of white preadipocytes to beige adipocytes through signaling pathway activation [39]. In vitro experiments proved that treatment with mulberry leaf was able to reduce lipid synthesis through the suppression of molecules that regulate differentiation from preadipocytes (PPARγ, C/EBPβ) by promoting factors related to lipolysis (i.e., CPT1, PPARα, AMPK, and HSL) [37,40,41]. The anti-inflammatory effects consisted of reducing pro-inflammatory cytokines, such as Interleukin-1 beta (IL-1β), Tumor necrosis factor (TNF-α), Inducible Nitric Oxide Synthase (iNOS), and Interleukin-6 (IL-6); enhancing antioxidant effects; and inhibiting free oxygen radical production, which all play key roles in adipocyte differentiation [40,42]. In addition, *Morus alba* has an antidiabetic effect. An experimental treatment with 1-DNJ significantly reduced blood glucose levels in hyperglycemia-induced mice [43], and this has been confirmed in humans [44,45,46]. In particular, according to a dose-finding study conducted in adult subjects for 12 weeks, 12 mg of mulberry DNJ was the minimum effective dose that could reduce postprandial hyperglycemia [47]. Moreover, in a recent clinical trial with healthy adults who received capsules containing 200 mg, 225 mg, and 250 mg of Reducose^®^ supplementation (i.e., a patented aqueous extract of white mulberry leaves standardized to contain 5% DNJ) ten minutes before the test meal, postprandial blood glucose and plasma insulin levels were lower compared to placebo [48].

In animal studies, *Morus alba* extract has been shown to improve both insulin resistance and blood glucose levels. In rats with diet-induced obesity, mulberry leaf extracts increased insulin receptor expression in muscle and adipose tissue [49]. Further studies confirmed that mulberry leaf extract could significantly reduce blood glucose levels in mice with high-fat diet-induced obesity [50,51]. In vitro studies described increased glucose uptake by the activation of the PI3-K signaling pathway, GLUT-4 translocation in rat adipocytes [52], and inhibition of *α*-glucosidase activity following the administration of mulberry leaf extract [53]. In addition, mulberry leaf polyphenols within the concentration range of 0.5–2 mg significantly suppressed the sodium/glucose 1 transporter (SGLT1), inhibiting glucose transport and reducing postprandial glucose uptake through the SGLT1-GLUT2 pathway [54].

In summary, emerging evidence highlights the promising role of specific nutraceutical compounds (such as Policaptil Gel Retard^®^ and white mulberry leaf extract) in supporting weight management and improving insulin sensitivity in pediatric obesity. These compounds exert multifaceted effects, including modulation of appetite, delay in carbohydrate and lipid absorption, enhancement of satiety hormones, and regulation of glucose and lipid metabolism. The main features and commonly used dosages of PGR and *Morus alba* are reported in Table 1.

### 3.3. Evidence for the Use of Pre- and Probiotics

The largest community of microbes in the human microbiota resides in the gut and, through a synbiotic relationship with the host, plays a role in maintaining health and metabolic homeostasis, including the production of an array of metabolites. Additionally, emerging evidence suggests that bacterial dysbiosis within the gut may be involved in the pathogenesis of obesity [55,56]. From a clinical point of view, there is great interest in determining if modulation of the gut microbiota is a viable strategy to manage obesity and improve metabolic health. Consumption of prebiotics, non-digestible food ingredients used by gut microorganisms, affects host physiology beneficially [55,56]. Microbial shifts in response to prebiotic intake are largely centered on changes in *Bifidobacterium* and *Lactobacillus*, two common genera that may be increased with the use of prebiotics. In different studies conducted in adults, the intake of prebiotics was associated with improved satiety feeling, postprandial glucose, and insulin concentrations [55]. Currently, there are few studies on prebiotics in overweight/obese children [55,56,57,58].

A study on 38 obese children (aged 7–12 years old) who consumed oligofructose-enriched inulin (8 g) once daily for 16 weeks presented a significant decrease in body weight z-score (3.1% decrease), body fat (2.4% decrease), trunk fat (3.8% decrease), serum triglyceride levels (19% decrease), and IL-6 serum levels (15% decrease) compared to children who received a placebo. This prebiotic caused significant changes in the bacterial community composition, notably increasing *Bifidobacterium* [55]. Also, oligofructose-enriched inulin improved children’s appetite ratings [57].

As to probiotics, a recent study involving 82 overweight and obese children evidenced that a 12-week supplementation with a multi-strain probiotic (i.e., *Lactobacillus salivarius* AP-32, *Lactobacillus rhamnosus* bv-77, and *Bifidobacterium animalis* CP-9) resulted in reduced BMI, lowered serum total cholesterol, low-density lipoprotein cholesterol (LDL-C), tumor necrosis factor-alpha (TNF-a), and leptin levels besides increased adiponectin and high-density lipoprotein cholesterol (HDL-C). The authors also suggested that the positive effects on lipids were related to *Lactobacillus* spp., while those on adiponectin to *Bifidobacterium animalis* [56,59]. Similarly, in adolescents with severe obesity, the consumption for 12 weeks of the oral probiotic “Visbiome^®^” containing multiple probiotic species (*Lactobacillus plantarum* DSM 24730, *Lactobacillus plantarum* DSM 24731, *Lactobacillus plantarum* DSM 24735, *Lactobacillus plantarum* DSM 24801, *Lactobacillus paracasei* DSM 24737, *Lactobacillus salivarius* DSM 24800, *Lactobacillus delbrueckii* DSM 25998, *Bifidobacterium animalis* DSM 24736, *Bifidobacterium breve* DSM 24732, and *Pediococcus pentosaceus* DSM 24734), improved the gut microbial composition and fasting glucose levels [60].

In rodents, the modulation of glucagon-like peptide 1 (GLP-1), glucose-dependent insulinotropic polypeptide (GIP), peptide YY (PYY), and ghrelin secretion through the use of fermentable dietary fibers might represent the link between microbial fermentation in the lower gut and the positive metabolic effects observed [61,62]. In adults, prebiotics would determine satiety by decreasing ghrelin and increasing intestinal plasma incretins [63,64]. A double-blind randomized controlled study on 10 healthy young adults aged 21–38 years who received supplementation with 16 g/day of prebiotics (Orafti Synergy1, a soluble fructan consisting of a mixture of glucosyl-(fructosyl)*n*-fructose and (fructosyl)*m*-fructose extracted from chicory root) for two weeks showed a significant increase in satiety, GLP-1, and PYY, and lowered postprandial plasma glucose levels [65]. Similarly, 10 young adults aged 21–39 years who supplemented for two weeks with 16 g/day of oligofructose showed a significant increase in satiety following breakfast and dinner and a reduction in hunger and food consumption compared to the placebo-treated group [66].

A randomized, double-blinded, placebo-controlled study on 155 obese children aged 7–15 years aimed to determine the effects of inulin supplementation on body composition and metabolic outcomes. Results showed that supplementing 13 g/day of inulin for six months significantly decreased BMI and improved body composition with an increase in fat-free mass [67].

It is also important to mention synbiotics, which are compounds that have both properties as prebiotics and probiotics, promote probiotic survival in the gastrointestinal tract, and may result in a better outcome when compared to prebiotics and probiotics separately [56]. A recent study in obese children (aged 8–18 yrs) who received a synbiotic capsule containing a combination of *Lactobacillus indicus* (6 × 10^9^ CFU) and *Lactobacillus coagulans* (6 × 10^9^ CFU) as probiotics and fructo-oligosaccharide as a prebiotic twice a day for 8 weeks observed a significant reduction in the waist–height ratio in the children receiving this combination compared to those receiving the placebo [68]. Another study pointed out that the group that received a synbiotic supplement once daily (*Lacticaseibacillus rhamnosus*, *Lactobacillus acidophilus*, *Bifidobacterium longum*, *Bifidobacterium bifidum*, and *Enterococcus faecium*, combined with fructo-oligosaccharides) for 12 weeks presented significant changes in weight, BMI, and waist circumference when compared to the placebo group [69].

The use of new-generation probiotics may also become part of prevention and treatment strategies for pediatric obesity. Recent studies have reported that the probiotic commensal bacteria strain *Hafnia alvei* (*H. alvei*), which produces the satietogenic peptide Casein-Like Protease B (ClpB), which mimics the effect of α-MSH, a key anorexigenic peptide involved in the regulation of appetite that stimulates in turn the release of PYY, reduces weight gain and adiposity in mouse models of obesity. A double-blind study on high-fat diet-induced hyperphagic ob/ob male mice, treated daily with 1.4 × 10^10^ CFU of *H. alvei* or placebo for 38 days, showed that the treatment resulted in decreased body weight, reduced fat mass gain, lowered food intake, and decreased glycemia, plasma total cholesterol, and alanine aminotransferase (ALT) [70]. In another study, oral administration of *H. alvei* HA4597 for 18 and 46 days in genetically obese mice (ob/ob) and high-fat-diet-fed obese mice (HFD), respectively, reduced body weight, fat mass gain, and food intake in hyperphagic and obese mice [71]. In 236 overweight adult subjects on a moderate hypocaloric diet who received two *H. alvei* HA4597 capsules (2.5 × 10^7^ CFU/capsule) per day, providing 100 billion bacteria per day, for 12 weeks, a significant improvement in body weight (3% reduction compared to initial body weight), reduction in hip circumference, and increased feeling of fullness were observed [72]. Therefore, current data are promising for the use of *H. alvei* as a probiotic for appetite and body weight management in overweight and obese individuals. However, studies are currently lacking in children.

In conclusion, pre- and probiotics show considerable potential as supportive interventions in the management of pediatric obesity and its metabolic complications. Their ability to modulate gut microbiota composition, enhance satiety, improve insulin sensitivity, and reduce inflammation offers a novel, non-invasive strategy to complement lifestyle modifications. Clinical studies in children have reported favorable outcomes on BMI, lipid profiles, glucose metabolism, and appetite regulation following supplementation with specific probiotic strains and prebiotic fibers. However, most evidence remains preliminary, and there is a need for standardized formulations, well-defined dosages, and larger, long-term pediatric trials. While promising, the incorporation of pre- and probiotics into clinical practice should be approached with caution until more robust data are available to support their efficacy and safety in the pediatric population.

## 4. Insulin Resistance

Obesity leads to insulin resistance, a risk factor for T2DM [73]. Insulin is released by pancreatic β-cells depending on the blood glucose level, binds to receptors expressed mainly in the liver cells, skeletal muscle, and adipose tissue, and allows glucose to enter cells for energy metabolism. When cells do not respond to insulin adequately, the blood glucose level rises, and pancreatic β-cells produce more insulin to compensate for the increase in circulating glucose, developing an insulin-resistant environment [73]. Insulin resistance is a complex condition influenced by various pathophysiological mechanisms. Among these, there is the accumulation of excessive free fatty acids (FFA) that leads to ectopic fat buildup [74]. This process results in an increase in reactive oxygen species (ROS) and chronic inflammation, both of which contribute to the development of insulin resistance. In obese individuals, inflammatory biomarkers such as C-reactive protein (CRP), TNF-α, and IL-6 are elevated and can directly inhibit insulin signaling [73]. Also, chronic inflammation reduces peroxisome proliferator-activated receptor-γ (PPAR-γ) expression with a further rise in FFA levels. Additionally, reduced hepatic insulin clearance leads to insulin receptor desensitization, decreased insulin release pulsatility, and less brown fat formation. Recognizing and understanding these mechanisms is crucial for taking preventive measures against T2DM [73].

### 4.1. Evidence for the Use of Vitamin D Supplementation

Extensive evidence supports the pivotal role of vitamin D in obesity and related cardiovascular complications [75]. Vitamin D levels are generally reported to be low in obese subjects due to different mechanisms: insufficient vitamin D intake, increased fat or muscle mass with increased deposits and reduced bioavailability, or different genotypes of vitamin D binding proteins or enzymes responsible for vitamin D metabolism [75]. Vitamin D is recognized to modulate glucose homeostasis by promoting insulin synthesis and glucose uptake both by the liver and peripherally [76]. Lower vitamin D levels have been associated with a significantly higher HOMA-IR index, BMI, and body fat percentage [77]. In a cross-sectional study of 64 obese children, vitamin D deficiency was prevalent in the subjects with lower insulin sensitivity and higher inflammatory markers [78]. Based on this evidence, several randomized clinical trials have studied the effects of vitamin D supplementation on body composition and insulin sensitivity. A randomized, double-blind, placebo-controlled trial involving 35 obese adolescents supplemented with 4000 IU/day of cholecalciferol showed significant improvements in HOMA-IR and quantitative insulin sensitivity check index (QUICKI) indexes with improved leptin/adiponectin ratio, all compatible with improved insulin sensitivity [79]. Similar results were reported in a further study of obese children who received 300,000 IU per week of vitamin D3 for 12 weeks [80]. However, data on obese pediatric patients are discordant. Regarding supplementation with 2000 IU/day, an increase in serum vitamin D concentration with beneficial effects on weight, BMI, and lipid balance without HOMA-IR index improvement was observed [81]. A one-time high dose of vitamin D2 (300,000 IU) did not modify insulin sensitivity in obese adolescents in another study [82]. Finally, a study in obese, vitamin D-deficient adolescents showed no significant change in the HOMA-IR index after supplementation with 100,000 IU of vitamin D3 once a month for three months [83]. Currently, we have not found any randomized clinical trials that explore the connection between vitamin D supplementation and insulin resistance in obese pediatric patients. Moreover, there is no consistency among studies about the doses and duration of vitamin D supplementation in the analyzed trials.

In conclusion, while vitamin D deficiency is commonly observed in obese children and has been associated with impaired insulin sensitivity and increased inflammatory markers, the evidence regarding the effectiveness of vitamin D supplementation in improving metabolic outcomes remains inconsistent. Some studies have reported improvements in insulin resistance and inflammatory profiles with high-dose vitamin D supplementation, while others have shown minimal or no significant effects. Most studies recommend a dose around 2000 IU per day for children with obesity. However, the variability in study designs, dosages, treatment durations, and population characteristics makes it difficult to draw definitive conclusions. Therefore, although correcting vitamin D deficiency is important for overall health, its role as a therapeutic agent for improving insulin sensitivity in pediatric obesity requires further investigation through well-designed, large-scale clinical trials.

### 4.2. Evidence for the Use of Anti-Inflammatory and Antioxidant Compounds, Macromolecules, and Inositols

Obesity is currently recognized as a low-grade chronic inflammatory disease [73]. This worsens insulin sensitivity and glucose homeostasis. Therefore, treatment with anti-inflammatory agents could be beneficial. A high percentage of obese children has been described to have a high-risk omega-3 index (percentage of eicosapentaenoic acid [EPA] and docosahexaenoic acid [DHA] with respect to total fatty acids on the erythrocyte membrane) compared with non-obese children. Also, the omega-3 index is associated with fasting insulin levels and the HOMA-IR index [84]. Thus, omega-3 is important in preventing metabolic comorbidities associated with obesity. Several studies have evaluated the effects of n3 long-chain polyunsaturated fatty acids (n3-LCPUFA) supplementation on insulin sensitivity in overweight and obese pediatric patients. Supplementation with 900 mg of n3-LCPUFA daily for one month resulted in decreased fasting insulin and HOMA-IR index, increased adiponectin levels, and reduced TNF-a and leptin levels [85]. Similar results were observed in a trial where omega-3 fatty acids were combined with antioxidant vitamins (130 mg of DHA, 25 mg of EPA, 200 µg of vitamin A, 1.25 µg of vitamin D, 2.5 mg of vitamin E, and 30 mg of vitamin C) [86]. Another study reported that omega-3 supplementation (1.8 g) has been beneficial in terms of weight loss, insulin sensitivity, and lipid profile compared with metformin (500 mg) in obese and insulin-resistant children and adolescents [87]. Two different randomized clinical trials reported that the combination of omega-3 PUFAs (1200 mg/day to 3000 mg/day, respectively) and lifestyle intervention significantly improved insulin sensitivity, dyslipidemia, and the adiponectin-leptin ratio compared with lifestyle intervention alone [88,89]. A randomized, double-blind clinical trial involving 366 adolescents with obesity found that supplementing with n3-LCPUFA for three months did not lead to any changes. Notably, a significant percentage of these children had extreme obesity, and the doses used (800 mg EPA + 400 mg DHA) may not have been sufficient to achieve the desired therapeutic effect [90].

As described above, Policaptil Gel Retard, a complex of polysaccharide macromolecules that slows carbohydrate absorption, reducing postprandial blood glucose peak and insulin levels, has positive effects on insulin sensitivity [28,29,30,31,32], and *Morus alba* improves glucose and insulin levels compared to placebo [47,48].

Gut dysbiosis, as previously described, is also related to insulin resistance; thus, probiotics may positively affect weight loss and insulin sensitivity, as reported in the previous paragraph [91]. Overall findings suggest that strains of *Bifidobacterium* can improve insulin sensitivity in obese subjects [92,93,94].

Antioxidant compounds, such as *curcumin*, may also have positive effects on insulin sensitivity. A randomized clinical trial was conducted with overweight and obese adolescent girls to evaluate the effects of curcumin supplementation on insulin resistance and lipid profiles. After a 10-week period of curcumin supplementation, a notable decrease in BMI, HDL, and triglyceride/high-density lipoprotein ratio was observed. However, there were no observed changes in the HOMA-IR index or blood glucose levels [95]. In contrast, another randomized trial reported that curcumin reduced insulin resistance by down-regulating resistin and fetuin-A [96]. Due to the limited number of studies and inconsistent findings, additional randomized clinical trials are required to establish the effectiveness of curcumin in enhancing insulin resistance in obese pediatric patients.

Myo-inositol (MYO) and D-chiro inositol (DCI) are two isomeric forms of inositol with insulin-sensitizing properties, acting on the intracellular insulin pathway. The effect of MYO and DCI supplementation on insulin sensitivity was evaluated in obese children with high fasting insulin levels and reported specifically that inositol (1100 mg of MYO, 27.6 mg of DCI, and 400 µg of folic acid daily intake) improved insulin sensitivity by means of an oral glucose tolerance test (OGTT) [97]. The effects of pinitol, a derivative of D-chiro-inositol, on glycemic control, insulin resistance, and adipokine levels were evaluated in a randomized, double-blind clinical trial. Participants received 1200 mg/day of pinitol for 12 weeks and reported a significant improvement in HbA1c, fasting blood glucose levels, and HOMA-IR index levels. However, there were no significant differences in adiponectin, resistin, FFA, and CRP levels [98]. Further research in obese adults reported no changes in insulin sensitivity, assessed using the gold standard euglycemic-hyperinsulinemic clamp after treatment. Additionally, there were no significant changes in fasting plasma glucose, HbA1c, insulin, FFA, lipoproteins, total cholesterol, LDL-C, HDL-C, and triglyceride levels, suggesting limited efficacy in improving glucose and lipid metabolism [99].

In vivo studies have shown the efficacy of treatment with inositols. In the db/db mouse model, having an obese phenotype in addition to hyperglycemia, dyslipidemia, and insulin resistance, MYO supplementation improved blood glucose levels, reduced insulin levels, and reduced triglyceride and total cholesterol levels, achieving results comparable to those obtained in metformin-treated mice. Moreover, it has been shown that MYO treatment could inhibit the differentiation of mesenchymal cells into adipocytes; however, MYO treatment had no effect on pancreatic β-cell proliferation and obesity [100]. DCI was assessed in mice on a high-fat diet and in vitro for its effects on fatty liver and insulin resistance. At a dose of 50 mg/kg/day, DCI reduced body weight in the mice, improved insulin resistance, and reduced levels of cytosolic phosphoenolpyruvate carboxykinase (PEPCK) and glucose-6-phosphatase (G6Pase), key enzymes in gluconeogenesis, besides reducing lipid accumulation in the liver. Furthermore, DCI inhibited the activation of PKCε (i.e., a diacylglycerol-activated protein that contributes to insulin resistance) and activated the phosphoinositide-3-kinase/activates protein B [PI3K/AKT] pathway, contributing to improved insulin signaling. Therefore, DCI was suggested as a potential treatment for hepatic steatosis and insulin resistance, with comparable efficacy to metformin [101].

The efficacy of the biological antioxidant alpha lipoic acid (ALA) in improving insulin sensitivity in overweight/obese patients has been investigated in several clinical trials. The evaluation of the effectiveness of treatment using ALA at a dosage of 600 mg twice daily after four weeks showed improved insulin sensitivity with a reported increase in both glucose utilization rate and insulin sensitivity index [102]. An eight-week clinical trial of 300 mg/day ALA supplementation in diabetic patients significantly reduced fasting glucose, postprandial blood glucose levels, and HOMA-IR index [103]. A placebo-controlled multicenter study investigated the effects of ALA (600 mg/day, 1200 mg/day, and 1800 mg/day) on insulin sensitivity over four weeks and found improved insulin sensitivity compared to placebo, with no significant differences among the doses used [104]. Another study used 1200 mg/day ALA supplementation for eight weeks and showed a significant reduction in BMI, weight and waist circumference but no changes in insulin resistance and oxidative stress [105].

In conclusion, the use of different nutraceuticals to improve insulin resistance in obese patients has shown promising results. Vitamin D supplementation at a dosage of 4000 IU per day was effective in improving insulin resistance. Lower dosages or one-time administration of high-dose vitamin D seems to have limited efficacy. Omega-3 fatty acids have shown positive results in the reduction in HOMA-IR index and inflammatory markers in obese pediatric patients from a dosage of 900 mg/day; however, higher doses of omega-3 would appear to be necessary to achieve effective results in adolescents with extreme obesity. Policaptil Gel Retard^®^ also improves glucose metabolism. Finally, both MYO and DCI and ALA are effective in improving insulin sensitivity, although some studies show different results.

The main nutraceuticals studied in patients with insulin resistance are summarized in Table 2.

## 5. Type 2 Diabetes Mellitus and Evidence for the Use of Nutritional Supplements

The chronic inflammatory state associated with obesity leads to a progressive deterioration of pancreatic beta-cell function with loss of insulin secretion [7,22,73,74,106]. The accumulation of fatty tissue increases the production of pro-inflammatory cytokines and fuels the systemic inflammatory state. This leads to reduced insulin signaling, loss of beta-cell function resulting in increased blood glucose levels, and the onset of diabetes mellitus. In particular, the accumulation of white adipose tissue in skeletal muscles leads to an increase in diacylglycerol (DAG), which activates protein kinase C, with subsequent reduction in glucose uptake in muscles, reduced insulin-induced glycogen synthesis, and increased gluconeogenesis in the liver. Inflammation also causes lipolysis, with an increased supply of glycerol and fatty acids to the liver, further increasing gluconeogenesis [106]. Saturated fatty acids increase the pro-inflammatory state by activating Toll-like receptor 4 (TLR4) and increasing endoplasmic reticulum stress by activating the Nuclear Factor kappa B (NF-kB) [74,106].

Many studies recommend diet and exercise interventions to improve metabolism and the inflammatory state in obese/overweight children [15,16,21,107].

As previously explained, gut microbiota plays an important role in metabolism and insulin regulation [106]. A randomized controlled trial evaluated synbiotic supplementation, combining one or more probiotics and prebiotics, on chronic inflammation and gut microbiota in obese adult patients with T2DM. No significant impact was reported on either inflammatory markers or glycemic control; however, gut microbiota composition was improved (increased number of bifidobacteria, lactobacilli, and organic acids in the feces) [108]. A randomized double-blind clinical trial evaluated the effects of a probiotic yogurt on glycemic control and antioxidant status in T2DM patients with a BMI up to 35 kg/m^2^. Consumption of 300 g/day of this yogurt containing *Lactobacillus acidophilus* La5 and *Bifidobacterium lactis* Bb12 for 6 weeks significantly reduced fasting blood glucose and HbA1c levels in association with increased superoxide dismutase and glutathione peroxidase activity [109]. Further studies showed a significant reduction in LDL-C levels, LDL-C to HDL-C ratio, and HbA1c levels [110]. Currently, studies evaluating the efficacy of probiotics in obese T2DM children are lacking.

Extra virgin olive oil has anti-inflammatory and antioxidant properties and is rich in monounsaturated fatty acids, phytosterols, hydrocarbons, and tocopherols. The effects of olive oil supplementation combined with a Brazilian diet (with foods such as rice, beans, lean meat, and vegetables) were evaluated in obese adult patients with T2DM. The study results showed that inflammatory markers decreased and adiponectin levels improved along with a reduction in fasting insulin levels, body weight, and BMI. However, further studies are needed, and no studies are available in pediatric patients [111].

Polyphenols have well-known and recognized properties. They improve glycemic control and prevent complications associated with diabetes through various mechanisms of action [112]. Polyphenols reduce food intake by producing cholecystokinin, which suppresses the sense of hunger, increases lipolysis by stimulating fatty acid beta-oxidation, and reduces lipid accumulation in adipocytes by activating AMPK [113]. In addition, they inhibit the proliferation of preadipocytes, favor their apoptosis, and inhibit pro-inflammatory cytokine production by inactivation of the NF-kB and mitogen-activated protein kinase (MAPK) pathways [113]. Moreover, polyphenols reduce the production of reactive oxygen species and protect pancreatic beta cells and the insulin receptor from oxidative injury, leading to increased insulin secretion and improved insulin resistance [112]. Finally, polyphenols have positive effects on gut microbiota by increasing the production of short-chain fatty acids (SCFAs) that are associated with improved insulin sensitivity [112,114]. The multicenter, randomized PREDIMED-Plus study, involving 6874 adult obese participants, analyzed the association between polyphenol consumption and the prevalence of T2DM, showing that the intake of specific polyphenols, such as catechins, proanthocyanidins, hydroxybenzoic acids, and lignans, was negatively associated with the prevalence of diabetes, being more effective in overweight individuals than in obese ones [115].

Resveratrol, a natural polyphenolic compound, supplemented at a dose of 1 g per day for 45 days, was associated with a reduction in fasting blood glucose and insulin levels, Hb1Ac, and improved HOMA-IR index in patients with T2DM [116]. Moreover, a randomized placebo-controlled clinical trial using 200 mg/day of resveratrol for 24 weeks also showed improved glycemic control [117].

Berberine is an isocholinic alkaloid extracted from *Berberis vulgaris* and other plants used in Chinese medicine. Berberine has been shown to modulate glucose and lipid metabolism, increase energy expenditure, and reduce body weight [118]. The effectiveness of berberine in improving insulin sensitivity and dyslipidemia was evaluated in adult T2DM patients who received a combination of berberine (500 mg twice a day) and silymarin (Silybum marianum, a 150 mg tablet twice a day) for 52 weeks and showed improved intestinal absorption, BMI, glycemic control, and lipid balance [119].

Decaffeinated green tea extract (GTE) is rich in the polyphenolic compound epigallocatechin gallate (EGCG). A randomized, double-blind clinical trial evaluated the effects of supplementing 856 mg/day EGCG (contained in 1500 mg GTE) in obese individuals with T2DM; after 16 weeks of treatment, no change in anthropometric measurements, fasting glucose, fasting insulin, HOMA-IR, HbA1c, and plasma lipoprotein levels was observed compared to the placebo group [120].

Curcuminoids are natural components extracted from turmeric belonging to the polyphenol family. A randomized, double-blind study reported significant reductions in fasting blood glucose levels, FFA and triglyceride levels, HbA1c, and HOMA-IR following a 300 mg/day intake of curcuminoids for three months in obese T2DM adult patients [121]. Co-administration of curcumin (500 mg/day) and piperine (5 mg/day), a black pepper extract that increases the bioavailability of curcumin, showed improved glycemic control in a similar population [122]. A recent double-blind, placebo-controlled study also showed that curcumin (1500 mg/day of curcumin for 12 months) was effective in reducing the atherogenic risk in obese patients with type 2 diabetes. Researchers observed a reduced pulse wave velocity (PWV), an indicator of arterial stiffness reduction, as well as reduced serum LDL-C and pro-inflammatory cytokines such as PCR, IL-1, IL-6, and TNF-α, all implicated in the pathogenesis of atherosclerosis, better glucose metabolism, decreased total and visceral body fat, and waist circumference [123]. Moreover, curcumin supplementation (1500 mg/day) has been demonstrated to be effective in preventing the development of T2DM in a prediabetic population [124]. It has been reported that a low zinc level is associated with reduced insulin sensitivity and increased inflammatory biomarkers [125]. Therefore, a study treated the patient with zinc (30 mg) or curcumin (500 mg) or both and reported a significant reduction in BMI in the group of zinc or zinc and curcumin combined, with improved fasting blood glucose levels, insulin, and HbA1c [126]. Moreover, single curcumin supplementation of 500 mg twice/day for prediabetic individuals improved insulin sensitivity and decreased fasting blood glucose levels and HOMA-IR [127]. In contrast, omega-3 fatty acids of 1000 mg twice/day in fish oil capsules were effective in reducing triglyceride levels. However, the co-administration of curcumin and omega-3 fatty acids did not show any additive effects [127].

Calanus finmarchicus oil supplementation has been studied in obese prediabetic subjects (2000 mg/day for 12 weeks) with reported improved glucose homeostasis and reduced fasting insulin levels and no change in Hb1Ac, thus at variance with the above-reported data [128].

Vitamin D supplementation has been reported to be effective in improving glucose metabolism. A dose of 50,000 IU/week of vitamin D3 for eight weeks resulted in a significant increase in SIRT1 (a glucose-regulating protein) and irisin (a myokine involved in energy metabolism) and a reduction in Hb1Ac; however, it was not significant [129]. In a further randomized clinical trial in adult obese patients, daily supplementation with 6000 IU of vitamin D3 for three months, followed by 3000 IU/day for another three months, and then 2200 IU/day for a further 6 months, showed no changes in body weight, fat mass, and waist circumference compared with the placebo group [130]. Therefore, there is a need for further studies in the pediatric population.

In conclusion, some nutraceuticals have shown efficacy in T2DM overweight/obese patients. In particular, curcumin supplementation was effective in improving glucose metabolism and reducing inflammation, as well as in preventing the progression to diabetes in prediabetic patients. The 1500 mg/day dosage of curcumin was also effective in reducing the atherogenic risk. Intake of omega-3 fatty acids at a 2000 mg/day dosage also showed some effects but with conflicting results. Resveratrol supplementation (1 g for a short period as well as 200 mg/day for several months) was effective in reducing insulin, blood glucose levels, HbA1c, and HOMA-IR index, and reduced inflammation and oxidative stress. Probiotics, which can be administered with yogurt, were also effective. However, more randomized clinical trials are needed, and studies in children are lacking.

The main nutraceutical agents that have been studied in T2DM patients are summarized in Table 3.

## 6. Dyslipidemia and Evidence for the Use of Nutritional Supplements

Obesity is a well-documented risk factor for dyslipidemia, as excess body fat, particularly visceral fat, is associated with changes in lipid metabolism, leading to high levels of LDL-C and triglycerides and reduced HDL-C levels. These changes contribute to the progression of atherosclerosis and the subsequent development of cardiovascular complications [131]. The prevalence of dyslipidemia among obese children has been reported to be up to 40% in a recent European cohort study [132] and to be 25–30% among Italian children [133]. The primary treatment approach involves implementing dietary strategies and promoting physical activity with the recommendation to monitor lipid assessment after the age of 6 years [21]. Interest in nutraceuticals for managing dyslipidemia is increasing, but research on their effects on mixed dyslipidemia is limited. Current European (ESC/EAS) guidelines recommend dietary supplements like plant sterols, fibers, polyunsaturated fatty acids, and red yeast rice alongside lifestyle changes, especially in obese adult individuals both on and off statin therapy [131].

Phytosterols improve lipid profiles by competing with cholesterol during intestinal absorption, lowering LDL-C levels [134]. Several studies have shown the benefits of phytosterols in managing dyslipidemia in children, especially with obesity. Daily supplementation of 1600 mg phytosterols associated with a specific cholesterol education program diet has been proven to reduce LDL-C levels [135], with similar results in other studies [136,137]. The 1600 mg/day of phytosterols positively affected plasma lipid levels and cholesterol metabolism [138]. Overall, phytosterols (up to 2 g daily) effectively improved lipid profiles in pediatric dyslipidemia, lowering plasma atherogenic LDL-C levels by up to 10% [139].

Vitamin D plays a significant role in regulating lipid metabolism by modulating gene expression in adipose tissue, reducing inflammation, and enhancing insulin sensitivity [80]. The benefits of vitamin D supplementation in improving insulin resistance and lipid profiles have been extensively reported in high-risk groups such as obese children and adolescents [80,140,141,142,143].

Probiotics are gaining attention as a potential treatment for dyslipidemia in obese children due to their positive effects on gut health, which may influence lipid profiles by reducing cholesterol absorption and enhancing bile acid production [144,145]. However, studies are still limited. Some studies describe that using synbiotics could lower inflammatory markers and improve metabolic parameters [146,147]. Nevertheless, more research is needed to confirm their effectiveness and optimize treatment strategies.

Polyunsaturated fatty acids (PUFAs), including omega-3 and omega-6, specifically eicosapentaenoic acid (EPA) and docosahexaenoic acid (DHA), positively impact lipid metabolism and inflammation in obese individuals as they decrease triglyceride production in the liver, lower LDL-C, and increase HDL-C levels by influencing key enzymes such as lipoprotein lipase and HMG-CoA reductase [148,149]. Additionally, PUFAs have effects on gene expression, as on the PPAR-α gene, which is key for fatty acid beta-oxidation [149]. In obese children, a substantial reduction in triglyceride levels and improved endothelial function after omega-3 supplementation and lifestyle change have been reported [89,150], and with hazelnut supplementation, lipid profiles have been improved [151]. The optimal dosage of PUFAs appears to be between 1 and 3 g of omega-3 per day. Observational and preliminary studies suggest the potential benefits of PUFA supplementation to improve lipid profiles [152,153]. However, the variability in results and methodological limitations highlight the need for further research. Also, rich in omega-3, *Portulaca oleracea* seeds have been reported to improve blood lipid levels in obese adolescents [154].

Dietary fiber supplementation has gained attention as a non-pharmacological strategy for managing dyslipidemia by reducing the intestinal absorption of cholesterol and bile acids, which helps lower LDL-C levels [155]. Two randomized, controlled, double-blind crossover studies reported that psyllium-enriched cereals significantly decreased total cholesterol and LDL-C in children with hypercholesterolemia [156,157]. Additionally, two non-crossover studies supported these findings, confirming that fiber supplementation could effectively reduce LDL-C levels [158,159]. However, research protocols are quite variable, and this highlights the need for further studies to reinforce existing evidence and examine the long-term effects of fiber use in children with dyslipidemia.

Some studies have examined the impact of antioxidants and ginger. Antioxidants are essential in neutralizing free radicals, which worsen dyslipidemia [160]. Interestingly, combining *ginger* with an anti-inflammatory diet significantly reduced cholesterol and triglyceride levels in obese kids [161].

In conclusion, studies support that phytosterols, vitamin D, probiotics, omega-3 fatty acids, dietary fibers, and ginger could positively impact dyslipidemia in obese children, improving lipid profiles and reducing inflammation. This underscores the value of integrating them into nutritional strategies for enhancing metabolic health and preventing obesity-related complications such as dyslipidemia.

The main nutraceutical agents that have been studied in patients with dyslipidemia are summarized in Table 4.

## 7. Metabolic Dysfunction-Associated Steatotic Liver Disease (MASLD) and Evidence for the Use of Nutraceutical Compounds

Metabolic dysfunction-associated steatotic liver disease (MASLD), which was previously named nonalcoholic fatty liver disease (NAFLD), is a condition characterized by hepatic steatosis and the presence of one or more of the metabolic risk factors [162]. MASLD encompasses a spectrum of conditions, ranging from simple steatosis (NAFLD) to non-alcoholic steatohepatitis (NASH), characterized by hepatocellular injury, ballooning, mixed lobular inflammation, pericellular-perisinusoidal fibrogenesis, and progression to fibrosis, cirrhosis, and hepatocellular carcinoma in a subset of cases [162,163]. There is a concerning rise in MASLD prevalence among children. A recent meta-analysis (2024) reported that MASLD/NAFLD affects about 13% of the general pediatric population [164], rising to 46–50% among obese children, thus being the most common chronic liver disease in developed countries. Obesity leads to unhealthy changes in adipose tissue, which is crucial for maintaining insulin sensitivity and preventing MASLD. Insufficient expansion of adipose tissue can cause chronic inflammation and fat accumulation in the liver [165]. Furthermore, the temporary physiological increase in insulin resistance during puberty may explain the higher prevalence of MASLD in obese children over 10 years of age [166]. Research suggests that methionine may promote hepatic lipogenesis, contributing to hepatic steatosis [167]. This leads to the hypothesis that reducing methionine concentrations through essential amino acid supplementation could enhance protein synthesis and mitigate hepatic fat accumulation.

The primary treatment for MASLD is lifestyle modification. However, in recent years, there has been a notable rise in interest in using nutraceuticals to improve liver steatosis. Various randomized controlled trials have shown that polyunsaturated fatty acids (PUFAs), such as DHA and EPA, can improve liver steatosis [168,169,170,171]. These fatty acids can lower blood triglycerides, provide anti-inflammatory effects, and reduce liver fat content [172,173]. Some researchers reported a significant reduction in liver fat with 250 mg of DHA, similar to higher doses, without changes in ALT levels [168,169,170]. However, data are still controversial.

Vitamin D has several effects on MASLD, as it reduces liver inflammation, improves lipid metabolism, modulates intrahepatic lipid accumulation, enhances insulin sensitivity, and influences liver fibrosis [165]. A randomized clinical trial of 100 children with biopsy-confirmed NAFLD found that those who received 2000 IU of vitamin D daily for six months showed significant improvements in steatosis and lobular inflammation and reductions in aspartate aminotransferase (AST) and alanine aminotransferase (ALT) levels compared to placebo [142]. In another study of 200 children, those receiving 5000 IU of vitamin D weekly showed a notable first- to second-grade improvement in fatty liver severity (*p* < 0.001) and greater reductions in ALT levels compared to controls [143]. However, the evidence for adults remains conflicting [97], suggesting the need for further clinical trials in both adults and children.

Most studies on antioxidants have focused on vitamin E supplementation, either alone [174,175], or combined with other supplements such as Hydroxytyrosol and vitamin C [176]. Vitamin E has antioxidant and anti-inflammatory properties and improves insulin resistance in adults [177,178]. Vitamin E at a dose of 800 IU/day for 96 weeks significantly reduced the NAFLD activity score (NAS) compared to placebo [174], with liver biopsies taken at the start and end of the study. Supporting this, the TONIC clinical trial observed a significant reduction in liver inflammation in the treatment group with vitamin E 800 IU/day but no differences in ALT [174]. Similar results were reported using lower 600 IU vitamin E daily supplementation for over 12 months [175]. Additionally, it seems that vitamin E combined with Hydroxytyrosol improves NAFLD in children and reduces inflammation and oxidative markers [176]. However, it was not possible to attribute the observed effects to any specific nutraceutical supplement. Finally, zinc supplementation has been shown to lower inflammatory markers and improve steatosis in overweight children [177], but this still requires further research.

MASLD is associated with changes in gut microbiota that cause inflammation and metabolic dysregulation [178]. *Enterococcus faecium* B6 produces tyramine, which can worsen liver metabolism and promote fat accumulation, contributing to MASLD progression in obese children. Targeting this microbiota may offer a therapeutic strategy for MASLD [179]. In a triple-blind placebo-controlled trial of 64 obese children with NAFLD, diagnosed by ultrasound, they received either a probiotic capsule or a placebo for 12 weeks. The probiotic group showed a significantly higher rate of normal liver ultrasonography and a lower incidence of high-grade fatty liver (*p* < 0.05) [180]. Another study of a probiotic mixture, VSL#3, which contains eight bacterial strains, has shown reduced steatosis and inflammatory markers in obese children with NASH [181]. However, further studies are needed to confirm these findings.

Other studies have explored the effects of different nutraceuticals on NAFLD and NASH. Such as a combination of DHA, choline, and vitamin E, resulted in significant improvements in liver fat and function tests [182], with similar findings for vitamin D [183].

In conclusion, although research on PUFA efficacy remains limited, most studies suggest benefits from omega-3, while findings on other nutraceuticals are inconsistent. Zinc and antioxidants, such as Hydroxytyrosol and vitamin E, might be of some help, and results related to the use of probiotics are promising, but data are yet insufficient. Finally, it remains unclear which specific nutraceuticals contribute to the positive outcomes, as combinations of compounds have often been used, although it could well be that combining multiple nutraceuticals may be more effective than using a single agent.

The main nutraceutical agents that have been studied in dyslipidemia patients are summarized in Table 5.

## 8. Conclusions

A comprehensive strategy that focuses on lifestyle changes—such as specific dietary adjustments and increased physical activity—is an effective method for addressing pediatric obesity and its associated metabolic issues. The combination of well-balanced dietary plans, like the Mediterranean or low-glycemic index diets, with organized physical exercise routines (especially involving aerobic and strength training) gives proven advantages. These strategies assist in reducing BMI, improving insulin sensitivity, and decreasing inflammatory markers, leading to a general improvement of metabolic health and possibly preventing obesity-related comorbidities.

Nutraceuticals should not replace dietary and lifestyle interventions, which remain the cornerstone of obesity management. Specific natural compounds should serve in addition to lifestyle modifications and have beneficial effects on glucose metabolism, lipid profiles, inflammation, and gut microbiota. We emphasize some nutraceutical products/dietary supplements that could be used in addition to the treatment of obesity and potentially could be early preventive measures, especially during childhood, a critical period for shaping long-term metabolic health outcomes. Compounds such as omega-3 fatty acids and vitamin D have demonstrated potential benefits in improving metabolic parameters. For example, omega-3 supplementation at doses of 2 g/day and vitamin D at doses of 2000 IU/day have been evaluated in obese pediatric populations, with reported improvements in lipid profiles, inflammatory markers, and insulin sensitivity. However, given the variability in study design, duration, and participant characteristics, these doses should not be generalized without clinical supervision.

As this work is a narrative rather than a systematic review, the possibility of selection bias cannot be ruled out. The current body of literature on nutraceutical use in pediatric populations is relatively sparse, with much of the evidence derived from adult studies or experimental models, which may not accurately represent pediatric physiology. Additionally, the included studies display significant heterogeneity in terms of methodology, sample size, intervention protocols, dosage, duration, and outcome parameters, which hampers the ability to draw direct comparisons or overarching conclusions. The generally short follow-up periods in pediatric trials further constrain our understanding of long-term efficacy and safety. Moreover, inconsistencies in dosing regimens and variations in the composition of nutraceutical products make interpretation more complex. Given the limited number of studies and conflicting results in the existing literature, we underscore the importance of a cautious and critical evaluation of the available evidence when considering the role of nutraceuticals in pediatric obesity and the need for systematic review. Lastly, the clinical application of these interventions in children with obesity remains limited by the lack of consensus guidelines, restricting their widespread adoption in practice.

Therefore, more large-scale, long-term, and pediatric-specific clinical studies are urgently needed. Future research should prioritize establishing standardized dosages, understanding age-specific responses, and evaluating long-term safety and efficacy to enable evidence-based and personalized approaches in clinical practice. Moreover, mechanistic insights into nutraceutical efficacy could be significantly enhanced through molecular-level analyses. Approaches such as genomic profiling or transcriptomic studies could help validate the modulation of inflammatory markers and metabolic pathways observed in clinical studies, thereby strengthening the biological plausibility and clinical relevance of these interventions.

In summary, the array of nutraceuticals mentioned in this review provides different strategies for managing comorbidities related to obesity, supporting lifestyle changes, and occasionally serving as alternatives to pharmacological therapies. Personalized, tailored, and safe interventions for pediatric patients are increasingly warranted.

## Figures and Tables

**Table 1:** Summary of nutraceutical compounds that have weight-lowering and positive metabolic effects.

Nutraceutical	Dose	Positive Effects	Limitations	References
*Policaptil Gel Retard^®^*	From 1450 mg/day to 2175 mg/day 20–30 min before meals	Improves body weight, glycemic control, and insulin sensitivity	Short follow-up studies and small sample sizes	[28,29,30,31,32]
*White mulberry leaves* (*Morus alba*)	Either 12 mg of DNJ or 250 mg of Reducose^®^ in adults	Improves insulin sensitivity, glycemic control, appetite control, and body weight. It has an inhibitory effect on the accumulation and synthesis of lipids	The majority of studies have been based on preclinical data. There are few studies in humans and no studies in children	[35,36,37,38,39,40,41,42,43,44,45,46,47,48,49,50,51,52,53,54]

**Table 2:** Summary of nutraceutical agents and compounds in individuals with insulin-resistance.

Nutraceutical	Dose	Positive Effects	Limitations	References
*Vitamin D*	4000 UI/day to 300,000 UI/week	Improved HOMA-IR and QUICKI, body weight, BMI, and lipid balance	Low-dose or one-time high doses are less effective	[79,80,81,82,83]
*Omega-3 fatty acids*	900 mg/day to 3000 mg/day	Improved insulin sensitivity, adiponectin-leptin ratio, and dyslipidemia	Ineffective in patients with extreme obesity	[85,86,87,88,89,90]
*Curcumin*	500 mg/day	Reduction in body mass index, improved lipid balance, and insulin resistance	Conflicting results regarding the effectiveness of curcumin in improving insulin resistance	[95,96]
*Myo-inositol and D-chiro inositol*	1100 mg/day MYO +27.6 mg/day DCI 1200 mg/day Pinitol	Improved insulin sensitivity in obese children Improved HbA1c, fasting blood glucose levels, and insulin sensitivity	Studies on pinitol supplementation differ and have been performed in adults only	[97,98,99]
*Alpha lipoic acid*	300 mg/day to 1800 mg/day	Reduction in fasting glucose and postprandial blood glucose levels, improved insulin sensitivity and body weight	Conflicting results on insulin resistance. No studies in pediatric cohorts	[102,103,104,105]

Note: HOMA-IR—Homeostatic Model Assessment for Insulin Resistance, BMI—body mass index, QUICKI—quantitative insulin sensitivity check index, MYO—Myo-inositol, DCI—D-chiro-inositol.

**Table 3:** Summary of nutraceutical supplements that have been studied in T2DM patients.

Nutraceutical	Dose	Positive Effects	Limitations	References
*Probiotics*	300 g/day of yogurt containing *Lactobacillus acidophilus* La5 and *Bifidobacterium lactis* Bb12	Reduction in fasting blood glucose, HbA1c, LDL-C, and LDL-C to HDL-C ratio	Studies are in adult patients only	[109,110]
*Extra virgin olive oil associated with Brazilian diet*	37.71 ± 12.23 mL/day	Reduction in inflammatory markers, fasting insulin levels, weight, and BMI	Single short-term study	[111]
*Resveratrol*	200 mg/day for 24 weeks–1 g/day for 45 days	Reduction in fasting glucose and insulin levels, Hb1Ac, and improved HOMA-IR index	Studies are in adults only	[116,117]
*Berberine + Sylmarin*	500 mg + 150 mg twice/day	Improved BMI, glycemic control, and lipid balance	Single study in adults	[119]
*Curcumin*	300 mg/day to 1500 mg/day	Reduction in fasting blood glucose levels, FFA, triglycerides, HbA1c, and HOMA-IR; reduction in atherogenic risk	Studies are in adults only	[121,122,123,124,125,126,127]
*Calanus finmarchicus oil*	2000 mg/day	Improved glucose homeostasis and reduction in fasting insulin levels	One single study in adult patients	[128]
*Vitamin D*	6000 UI/day–50,000 UI/week	Increase in SIRT1 and irisin and decrease in Hb1Ac	Low effectiveness in reducing body weight. Studies are in adults only	[129,130]

Note: HOMA-IR—Homeostatic Model Assessment for Insulin Resistance, BMI—body mass index, LDL-C—low-density lipoprotein cholesterol, HDL-C—high-density lipoprotein cholesterol, FFA—free fatty acids, SIRT1—sirtuin 1.

**Table 4:** Summary of nutraceutical compounds that have been studied in patients with dyslipidemia.

Nutraceutical	Dose	Positive Effects	Limitations	References
*Phytosterols*	1.6 g/day to 2 g/day	Reduction in TC, LDL-C, and improved lipoprotein composition	Short-term follow-ups and limited sample sizes	[134,135,136,137,138,139]
*Vitamin D*	2000 IU/day to 50,000 IU/day	Improved LDL-C, triglycerides, and insulin resistance	Studies using different doses	[140,141,142,143]
*Probiotics*	1 billion CFU/day to 2 billion CFU/day	Reduction in inflammatory markers	Short-term follow-ups and limited formulations	[146,147]
*Omega-3 fatty acids*	1 g/day to 1.2 g/day	Reduction in triglycerides, improved lipid profile, increased unsaturated fatty acids	Short-term follow-ups	[150,151,152,153]
*Portulaca oleracea*	10 g/day	Reduction in TC and LDL-C	Limited sample size and based on preliminary results	[154]
*Dietary fiber*	3 g/day to 6 g/day	Reduction in TC and LDL-C	Short-term follow-ups with variable compliance in children	[156,157,158]
*Ginger*	2 g/day	Reduction in TC and inflammatory markers	Limited sample size	[161]

Note: LDL-C—low-density lipoprotein cholesterol, TC—total cholesterol.

**Table 5:** Summary of nutraceutical compounds that have been studied in patients with MASLD.

Nutraceutical	Dose	Positive Effects	Limitations	References
*Vitamin D*	2000 IU/day	Reduced liver steatosis and improvement of ALT, AST	Short-term follow-ups and study design limitations	[142,143]
*Probiotics*	1 billion CFU/day to 10 billion CFU/day	Reduced liver steatosis and histological improvement of NASH	Short-term follow-ups	[180,181]
*Omega-3 fatty acids*	600 mg/day to 1 g/day	Reduced liver fat and cardiometabolic improvement	Short-term follow-ups and non-uniform dosage	[168,169,170,171]
*Vitamin E*	400 IU/day to 800 IU/day	Reduction in ALT, AST, and insulin resistance	Short-term follow-ups and study design limitations	[174,175]
*Zinc*	20 mg/day	Reduced inflammatory markers and improved NASH	Short-term follow-ups and small study groups	[177]
*Combination of Hydroxytyrosol and Vitamin E*	Vitamin E: 600 IU/day to 800 IU/day Hydroxytyrosol: 10 mg/day to 30 mg/day	Reduction in inflammatory and oxidative markers	Conflicting results regarding the effect of each component separately and in combination	[176]

Note: ALT—alanine aminotransferase, AST—aspartate aminotransferase, NASH—non-alcoholic steatohepatitis.

## Abbreviations

The following abbreviations are used in this manuscript:

1-DNJ	1-deoxynojirimycin
AAP	American Academy of Pediatrics
ALA	alpha lipoic acid
ALT	alanine aminotransferase
AST	aspartate aminotransferase
AMPK	AMP-activated protein kinase
BMI	body mass index
CRP	C-reactive protein
DCI	D-chiro inositol
DAG	diacylglycerol
DHA	docosahexaenoic acid
EPA	eicosapentaenoic acid
FFA	free fatty acids
G6Pase	glucose-6- phosphatase
GLP-1	glucagon-like peptide 1
GIP	glucose-dependent insulinotropic polypeptide
HbA1c	glycosylated hemoglobin A1c
HCC	hepatocellular carcinoma
HDL-C	high-density lipoprotein cholesterol
HOMA-IR	Homeostasis Model Assessment for Insulin Resistance
IL-1	interleukin 1
IL-6	interleukin 6
LDL-C	low-density lipoprotein cholesterol
MAPK	mitogen-activated protein kinase
MASLD	metabolic dysfunction-associated steatotic liver disease
MetS	metabolic syndrome
MYO	Myo-inositol
n3-LCPUFA:	n3 long-chain polyunsaturated fatty acids
NAFLD	nonalcoholic fatty liver disease
NASH	non-alcoholic steatohepatitis
NF-kB	Nuclear Factor kappa B
OGTT	oral glucose tolerance test
PEPCK	phosphoenolpyruvate carboxykinase
PGR	Policaptil Gel Retard^®^
PPAR-γ	peroxisome proliferator-activated receptor-γ
PUFAs	polyunsaturated fatty acids
PYY	peptide YY
QUICKI	quantitative insulin sensitivity check index
ROS	reactive oxygen species
SCFA	short-chain fatty acids
SGLT1	sodium/glucose 1 transporter
SIRT1	sirtuin 1
T2DM	type 2 diabetes mellitus
TLR4	toll-like receptor 4
TNF-α	tumor necrosis factor-alpha
WHO	>World Health Organization
